# Dopamine Transporter Genetic Variants and Pesticides in Parkinson’s Disease

**DOI:** 10.1289/ehp.0800277

**Published:** 2009-02-22

**Authors:** Beate R. Ritz, Angelika D. Manthripragada, Sadie Costello, Sarah J. Lincoln, Matthew J. Farrer, Myles Cockburn, Jeff Bronstein

**Affiliations:** 1 Department of Epidemiology and; 2 Department of Environmental Health Sciences, Center for Occupational and Environmental Health, UCLA School of Public Health, University of California at Los Angeles, Los Angeles, California, USA; 3 Department of Neurology, UCLA School of Medicine, University of California at Los Angeles, Los Angeles, California, USA; 4 Department of Environmental Health Sciences, School of Public Health, University of California at Berkeley, Berkeley, California, USA; 5 Division of Neurogenetics, Department of Neuroscience, Mayo Clinic, Jacksonville, Florida, USA; 6 Department of Preventive Medicine, Keck School of Medicine, University of Southern California, Los Angeles, California, USA

**Keywords:** dopamine transporter, gene-environmental interactions, occupational and environmental exposures, Parkinson’s disease, pesticides

## Abstract

**Background:**

Research suggests that independent and joint effects of genetic variability in the dopamine transporter (*DAT*) locus and pesticides may influence Parkinson’s disease (PD) risk.

**Materials:**

Methods: In 324 incident PD patients and 334 population controls from our rural California case–control study, we genotyped rs2652510, rs2550956 (for the *DAT* 5′ clades), and the 3′ variable number of tandem repeats (VNTR). Using geographic information system methods, we determined residential exposure to agricultural maneb and paraquat applications. We also collected occupational pesticide use data. Employing logistic regression, we calculated odds ratios (ORs) for clade diplotypes, VNTR genotype, and number of susceptibility (A clade and 9-repeat) alleles and assessed susceptibility allele–pesticide interactions.

**Results:**

PD risk was increased separately in *DAT* A clade diplotype carriers [AA vs. BB: OR = 1.66; 95% confidence interval (CI), 1.08–2.57] and 3′ VNTR 9/9 carriers (9/9 vs. 10/10: OR = 1.8; 95% CI, 0.96–3.57), and our data suggest a gene dosing effect. Importantly, high exposure to paraquat and maneb in carriers of one susceptibility allele increased PD risk 3-fold (OR = 2.99; 95% CI, 0.88–10.2), and in carriers of two or more alleles more than 4-fold (OR = 4.53; 95% CI, 1.70–12.1). We obtained similar results for occupational pesticide measures.

**Discussion:**

Using two independent pesticide measures, we *a*) replicated previously reported gene–environment interactions between *DAT* genetic variants and occupational pesticide exposure in men and *b*) overcame previous limitations of nonspecific pesticide measures and potential recall bias by employing state records and computer models to estimate residential pesticide exposure.

**Conclusion:**

Our results suggest that *DAT* genetic variability and pesticide exposure interact to increase PD risk.

Parkinson’s disease (PD) is a chronic, progressive, neurodegenerative movement disorder characterized by a loss of the neurotransmitter dopamine. The dopamine transporter (DAT) is responsible for the reuptake of dopamine into presynaptic neurons, which terminates dopamine neurotransmission and thus plays a central role in the spatial and temporal buffering of released dopamine and its recycling (Uhl 2003). In drug-naive patients with PD, the levels of DAT protein are dramatically reduced (Lee et al. 2000). Human genetic variability in *DAT*, the gene encoding DAT (*SLC6A3*, GeneID 6531; National Center for Biotechnology Information 2009) has been associated with disorders and behaviors thought to be influenced by dopamine signaling such as attention-deficit hyperactivity disorder (Yang et al. 2007), bipolar disorders (Greenwood et al. 2006), and smoking cessation (Stapleton et al. 2007). *DAT* gene deletion in the mouse results in hyperactivity (Gainetdinov 1997; Giros et al. 1996), and when tyrosine hydroxylase is also inhibited, these animals show physical behaviors (e.g., akinesia, rigidity, tremor) that phenotypically resemble the motor symptoms of PD (Sotnikova et al. 2006).

Given its central role in dopaminergic neurotransmission, *DAT* has been studied as a candidate gene for PD but with largely equivocal results (Bagade et al. 2008). However, PD is considered to have a multifactorial etiology, and further insight into the role of *DAT* gene variants on PD risk might be obtained from investigations of gene–environment interactions. Kelada et al. (2006) first reported on the combined effect of *DAT* genetic variability and occupational pesticide exposure. Their gene–environment interaction investigation was prompted by the hypothesis that DAT can selectively carry certain neurotoxicants into dopaminergic neurons; for example, 1-methyl-4-phenylpyridinium ion (MPP^+^), a complex I mitochondrial poison, has devastating effects on the basal ganglia due to its selective uptake into dopaminergic neuronal terminals via DAT (Gainetdinov 1997; Javitch et al. 1985; Langston et al. 1984). Mitochondrial toxins, including pesticides, are thought to play a role in the etiology of PD (Przedborski and Ischiropoulos 2005). Initially, attention focused on the herbicide paraquat because of its structural similarity to MPP^+^. In rodent models, paraquat induces a number of parkinsonism-like phenotypes, including motor degeneration with progressive reduction in dopamine, and α-synuclein–immunopositive neuronal pathology, especially when administered together with the fungicide maneb (Norris et al. 2007; Thiruchelvam et al. 2000, 2003). Epidemiologic studies have now implicated a number of different pesticides, including insecticides, fungicides, and herbicides, in the etiology of PD (Elbaz and Tranchant 2007).

In an observational study, the finding that an environmental agent’s impact is modified by a subject’s genetic variability may be considered to provide more compelling evidence of a disease association. However, gene–environment interaction analyses for pesticides and PD have been rare, and replications are almost nonexistent (Deng et al. 2004; Elbaz et al. 2004; Hancock 2008; Kelada et al. 2006). By design, our case–control study of incident PD in rural counties in central California provides a unique opportunity to investigate both self-reported occupational pesticide exposure and computer-modeled residential pesticide exposure, the latter using data collected during 30 years of state-mandated pesticide reporting and integrated into a geographic information system (GIS) approach. We based our occupational exposure measure on “any kind” of occupational pesticide exposure, identical to the approach taken by Kelada et al. (2006), to replicate the previous analysis as closely as possible. We did not attempt to extend the occupational exposure analysis to selected agents because such data were too sparse for an interaction analysis and were more likely to be biased because of subject recall. For residential exposures derived from our GIS model, we concentrated on estimating paraquat and maneb exposures because *a*) it has been hypothesized that paraquat is transported into dopamine cells by DAT (Shimizu et al. 2003), although this notion has been challenged (Richardson et al. 2005); *b*) animal data suggest that multiple paraquat applications or exposure to both maneb and paraquat are needed to cause PD-like pathology in these models (Thiruchelvam et al. 2000); and *c*) paraquat and maneb are both commonly used by the agricultural businesses in the studied counties, and only four subjects considered residentially exposed to maneb were unexposed to paraquat, so analysis of maneb alone would be inconclusive.

We have tested the gene–environment hypothesis that *DAT* genetic variants alone and in combination with occupational or residential pesticide exposures increase susceptibility to PD. We aimed to replicate the findings of Kelada et al. (2006) using our occupational pesticide measures. In addition, with our GIS model we hoped to overcome previous limitations of *a*) nonspecific pesticide exposure assessment and *b*) potential biases introduced by subject recall of self-reported pesticide exposures.

## Materials and Methods

All procedures described have been approved by the University of California at Los Angeles (UCLA) Human Subjects Committee, and we obtained informed consent from all participants.

### Subject recruitment

Using a population-based approach, we recruited cases and control subjects in three central California counties: Fresno, Tulare, and Kern. Case definition and recruitment criteria have been described in detail elsewhere (Kang et al. 2005). Briefly, with the help of neurologists practicing in or near this region, large medical groups, and public service announcements, we contacted patients with incident PD residing in these counties. For the analyses presented here, we enrolled cases from early 2001 until July 2007; cases were examined by UCLA movement disorder specialists at least once, and confirmed as having clinically “probable” or “possible” PD (Kang et al. 2005).

We had initial contact with 1,124 potential cases and determined 600 to be ineligible: 51 had never received a PD diagnosis, 133 did not live in the tricounty area or had not lived in California for 5 years or more, 394 had been diagnosed with PD more than 3 years before recruitment, and 22 patients were too ill to participate in our study. Another 88 potential cases could not be examined or interviewed (46 withdrew, and 42 were too ill, had died, or had moved away before the scheduled appointment). A total of 436 eligible cases were examined on multiple occasions as necessary by our UCLA movement disorder specialists to confirm a diagnosis of clinically probable or possible PD using well-established, stringent diagnostic criteria (Kang et al. 2005). Of the subjects examined, we excluded 95 because they received diagnoses such as parkinsonism from other causes, 12 did not provide DNA samples or the samples they provided failed during genetic analyses, and five did not provide all necessary data. Ultimately, 324 cases contributed risk factor and genetic data to this analysis.

For the first year of recruitment, we identified control subjects > 65 years of age from Medicare lists, but Health Insurance Portability and Accountability Act (HIPAA) implementation prohibited the continued use of Medicare lists in subsequent years. Thus, we recruited most control subjects (> 75%) from randomly selected residential parcels identified from publicly available tax-collector records providing addresses for all zoned living units in Kern, Fresno, and Tulare Counties. For eligibility screening purposes, we had initial mail and/or phone contact with 878 potential control subjects. Control eligibility criteria included *a*) not having PD, *b*) ≥ 35 years of age, and *c*) currently residing in one of the three counties and having lived in California for at least 5 years before our screening. Only one person per parcel unit was allowed to enroll. We found 221 potential population control subjects were not eligible: 170 were too young, 45 were terminally ill or according to respondents recently deceased, and six did not primarily reside in the tricounty area. Of the 657 eligible population controls, 285 declined participation, were too ill, or had moved out of the area before interview; we enrolled 372 (57%), and for 334 we successfully genotyped for the *DAT* variants and had all necessary data for the analyses.

### Pesticide exposures assessment

We conducted telephone interviews to obtain demographic and risk factor information including detailed occupation and residential histories. We estimated pesticide exposures in the ambient residential environment resulting from applications to agricultural crops by employing a GIS computer model, which combined geocoded lifetime residential histories, California pesticide use reporting data, and land use maps. A technical and more detailed discussion of our geocoding and GIS-based approaches is provided elsewhere (Costello et al. 2009; Goldberg et al. 2008). Here we briefly summarize the data sources and the exposure modeling process. Pesticide-use reports (PURs) are recorded by the California Department of Pesticides Regulations for all commercial applications of pesticides, including agricultural applications. Each PUR record includes the name of the pesticide’s active ingredient, the poundage applied, the crop and acreage of the field, the application method, the date of application, and a PUR locator, which can be linked to the Public Land Survey System, a nationwide grid that parcels land into sections of approximately 1 square mile (640 acres). To more precisely locate the pesticide application, we combined information from land use maps with that from PUR, as described in detail elsewhere (Rull and Ritz 2003). For each pesticide listed in the PURs, we created a year-specific average exposure estimate for each subject. First, we summed pounds of pesticide per year per acre applied within a 500-m radius buffer of each residence (Chester and Ward 1984; MacCollom et al. 1986; McElroy et al. 2003). Then, for each subject, we calculated a study period average by summing the year-specific averages for each chemical from 1974 to 1999 and dividing that sum by 26, the total number of years in the relevant time period.

For residential pesticide exposure, we categorized subjects as “highly exposed” if they had a study period average that fell above the pesticide-specific median value. Subjects exposed to both maneb and paraquat above the median value we considered highly exposed for this analysis; all other subjects we considered “low” or “unexposed.” We were unable to derive residential pesticide exposure estimates for 19 subjects (9 PD and 10 control subjects) because they had not lived in one of the three counties during the period for which PUR pesticide data were available (1974–1999). We assumed that these individuals had not been exposed and performed sensitivity analyses excluding these individuals. The average amount of maneb and paraquat applied near homes of study subjects was relatively stable throughout the time window of 1974–1999, except that annual paraquat exposure increased in the late 1990s.

Additionally, we created estimates of occupational exposure to pesticides for subjects who had held jobs in the agricultural sector, assigning them into categories of “likely exposed” when they reported pesticide handling and applications or fieldwork, and “possibly exposed” when reporting managerial, produce processing, and other nonfield farmwork; all others we considered “not occupationally exposed”(Young et al. 2004). Only 15 (9%) of the 167 subjects considered occupationally exposed were also considered residentially highly exposed to both maneb and paraquat.

### Selection of single nucleotide polymorphisms and laboratory methods for genotyping

From 22 single nucleotide polymorphisms (SNPs) found in the 5′ region of *DAT*, Kelada et al. (2005) identified eight haplotypes that can be grouped into two evolutionary clades, A and B (Kelada et al. 2006). Although these authors used six SNPs to identify the haplotypes for categorization into A and B clades, four of these provide redundant information because they are in linkage disequilibrium. Only two SNPs, rs2652510 (−2315) and rs2550956 (−2296), are needed to define the 5′ clades, and were genotyped along with the 3′ variable number of tandem repeats (VNTR). We considered the 5′ A clade and the 3′ VNTR 9-repeat susceptibility alleles; thus, an individual could have a maximum of four susceptibility alleles: two copies of the A clade 5′ region and two copies of the 9-repeat 3′ VNTR.

Participants provided blood or buccal samples for genetic analyses. Blood/buccal samples were stored and processed at the UCLA Biologic Specimen Core Facility. All genotyping was done at the Mayo Clinic in Jacksonville, Florida. *DAT* SNPs rs2652510 and rs2550956 were amplified from 20 ng of genomic DNA using Applied Biosystems, Inc. (ABI) Taqman polymerase chain reaction (PCR) Mastermix (assay IDs C_3284852_10 and C_3284851_10, respectively) (ABI, Foster City, CA). PCR products were analyzed on an ABI 7900 instrument using SDS version 2.2.2 allelic discrimination software (ABI). The 40 bp 3′ VNTR of *DAT* was amplified using FAM-labeled primers (Vandenbergh et al. 1992). PCR products analyzed on an ABI 3730 automated sequencer and the number of repeats were determined using GeneMapper 4.0 (ABI).

### Statistical methods

We confirmed Hardy–Weinberg equilibrium in PD cases and control subjects separately for all polymorphisms. We estimated main effects of genotype and diplotype frequencies, relying on logistic regression analyses to calculate odds ratios (ORs) and 95% confidence intervals (CIs) adjusted for potential confounders including sex, smoking status (ever/never), age (continuous), race (white, black, Latino, Asian, and Native American), county (Kern, Tulare, and Fresno), occupational pesticide use (unexposed, possibly exposed, likely exposed), and residential pesticide exposure to paraquat and/or maneb (none, low, high). We also categorized individuals by the number of susceptibility alleles.

We assessed interactions between the number of *DAT* susceptibility alleles and exposures to both pesticide measures by employing stratified analyses and by introducing interaction terms into logistic models. The former can be directly compared with Kelada et al. (2006); estimation of ORs and CIs from logistic regression models, with interaction terms, was not appreciably different (data not shown).

We also performed main effect analyses stratified by sex and by age at onset (≤ 60, > 60 years of age at diagnosis for patients, or by interview date for control subjects) and performed sensitivity analyses evaluating Caucasians only to account for the possibility of population stratification in our mixed ethnicity group. SAS version 9.1 (SAS Institute Inc., Cary, NC) was used to perform unconditional logistic regression analyses.

## Results

Study participants were predominantly Caucasian and older than 60 years of age and did not report a family history of parkinsonism (Table 1). PD patients were slightly older, more often male, and less educated than were control subjects. They were also more likely to have never smoked cigarettes or to have quit smoking and to have been pesticide exposed. High residential exposures to both paraquat and maneb between 1974 and 1999 increased the risk of PD more than 2-fold (adjusted OR, 2.32; 95% CI, 1.23–4.40), and occupational exposure increased risk of PD by approximately 50% (males: adjusted OR, 1.56; 95% CI, 0.95–2.56).

Our data suggest a moderate increase in the risk of PD separately for both the homozygous AA diplotype of the 5′ clade (OR, 1.66; 95% CI, 1.08–2.57) and for the 9/9 3′ VNTR (OR, 1.86; 95% CI, 0.96–3.57; Table 2). Assessing the cumulative effect of susceptibility alleles (defined as the 5′ A clade and the 3′ VNTR 9-repeat), we found a 50% increase in risk for carriers of more than two *DAT* susceptibility alleles (OR, 1.50; 95% CI, 1.00–2.25; Table 3). Furthermore, our data also suggested an allele dosage effect with increasing number of susceptibility alleles (2 vs. 0 susceptibility alleles: OR, 1.26; 95% CI, 0.82–1.95; 3 vs. 0 alleles: OR, 2.18; 95% CI, 1.17–4.06; 4 vs. 0 alleles: OR, 3.61; 95% CI, 0.93–14.1). All results were similar when we restricted our analyses to Caucasians only or when we stratified by sex or age at PD onset (≤ 60 and > 60 years).

When stratifying by levels of residential exposure to both maneb and paraquat, we found that high exposure increased risk almost 3-fold in subjects who carried one *DAT* susceptibility allele and as much as 4.5-fold in carriers of two or more susceptibility alleles (OR, 4.53; 95% CI, 1.70–12.09). Yet, in those subjects with little or no residential exposure to these pesticides, we observed no indication of increase in risk with susceptibility allele carrier status or increasing number of susceptibility alleles (Table 4). Restricting analyses to Caucasians only, stratifying by age at onset, or stratifying by sex did not change these results. As in the previous study by Kelada et al. (2006), we examined the influence of occupational pesticide exposure in males only (*n* = 347). After adjusting for high residential exposure as well as the other relevant factors, we observed a 2-fold and almost 3-fold increase in PD risk among likely pesticide-exposed carriers of one and of two or more *DAT* susceptibility alleles, respectively, but no association among the occupationally unexposed regardless of the number of *DAT* susceptibility alleles (Table 5).

## Discussion

Our population-based case–control study of PD conducted in a California population heavily exposed to pesticides replicates and extends evidence for an association between *DAT* variants and PD (Kelada et al. 2006) and highlights possible interactions of disease-associated *DAT* susceptibility alleles and pesticide exposure (Tables 2–5).

All eight major *DAT* 5′ region haplotypes are part of two evolutionary clades (clades A and B) (Kelada et al. 2006). *In vitro* study of the six common haplotypes indicated that the two most prevalent haplotypes of clade A have 40–50% higher luciferase activity compared with the two most prevalent haplotypes of clade B (Kelada 2005). A caveat is that *in vitro* gene reporter assays are often dependent on cell type, transcription factor, and chromatin context. In contrast, direct *in vivo* human imaging of DAT binding and gene expression studies in postmortem brains suggest that B clade haplotypes are associated with higher DAT levels (Drgon et al. 2006). Conflicting functional results are difficult to reconcile, but, in agreement with Kelada et al. (2006), we subscribe to the view that *in vivo* measures are more likely to be representative of the true physiologic picture. Thus, our data suggest that lower DAT function/levels due to clade A may increase susceptibility to PD and, by extension, that DAT levels affect PD risk only in those subjects who are pesticide exposed.

Thus, the combination of these genetic associations and previous *in vivo* functional observations appears to contradict the long-held belief that DAT provides a gateway for MPP^+^, paraquat, and maneb and thus potentiates their neurotoxic effects in dopaminergic neurons (Edwards 1993). To date, there remains no conclusive evidence that pesticides enter dopaminergic neurons via DAT; in fact, a recent study showed DAT to be an unlikely transporter for paraquat (Richardson et al. 2005). Indeed, the selective vulnerability underlying loss of dopaminergic neurons remains enigmatic. Placing the observed and now confirmed gene–pesticide interaction association in biologic context likely requires further understanding of the roles of DAT, paraquat and maneb, and the toxic mechanisms they exert on neurons. Paraquat’s toxic action is often attributed to reduction–oxidation cycling that generates reactive oxygen species (Przedborski and Ischiropoulos 2005). For maneb, the neurotoxic mechanism may be mediated by ubiquitin-proteasome system inhibition (Wang et al. 2006; Zhou et al. 2004).

Most important in our study, risk of PD seems to depend on whether subjects are exposed to pesticides. We observed little indication that *DAT* susceptibility allele(s) affect risk in those unexposed to agriculturally applied maneb and paraquat or occupationally (albeit self-reported) exposure to any type of pesticide. For occupationally exposed males, we estimated an almost 3-fold increase in risk for those carrying two or more susceptibility alleles and a 2-fold increase in risk for those with only one allele, compared with those not carrying *DAT* susceptibility alleles. Our results thus replicate a strong gene–pesticide interaction (> 5-fold risk increase; Table 5) previously reported for occupationally pesticide-exposed males (Kelada et al. 2006). Moreover, we employed our GIS-derived, record-based residential pesticide exposure estimates for maneb and paraquat and found that highly exposed subjects with one *DAT* susceptibility allele have an estimated 3-fold increase, and subjects with two and more alleles a 4.5-fold increase, in risk of PD compared with those with no *DAT* susceptibility alleles. There was little or no indication of a *DAT* susceptibility allele association in subjects with no or low residential pesticide exposure as estimated by our GIS model (Table 4).

A limitation of our study is the relatively small sample size for some strata of our gene–environment interaction analysis. This may affect the informativeness of the data and the interpretability of results. In research of rare diseases, such as PD, sample size is always an issue for gene–environment interaction studies. However, collaborations and data pooling efforts to increase sample size are usually limited by the need to arrive at comparable and valid measures of exposures, in addition to identifying a large enough exposed population in each study.

A primary strength of our study is the estimation of residential pesticide exposure using a GIS-based computer model and not subjects’ self-reports; therefore, our residential estimates are unlikely to be biased by differential recall. Some nondifferential exposure misclassification possibly attenuates effect estimates, and we encountered some missing or incomplete address information and geocoding problems in our GIS approach. Residential pesticide exposure will also depend on wind patterns at the time of application, open windows, and the likelihood of tracking dust and pesticide residues into homes. There is no obvious reason why subjects would have participated in our study based on a history of living near agricultural plots, and most rural residents might not know what was applied on fields near their homes in the past decades. It is even less likely that subjects would be able to self-select themselves according to both genotype and pesticide exposure. A strength of our study is that, in contrast to most previous occupational and environmental epidemiologic studies of PD, all of our diagnoses were clinically confirmed by one or more examinations by UCLA movement disorder specialists, so disease misclassification is likely to be minimal.

Our residential pesticide estimates are unique in the field of epidemiologic exposure assessment, and our rationale for their use in this study is strong. In this region of California, our two pesticides of particular interest, maneb and paraquat, are both applied on common crops such as potatoes, dry beans, and tomatoes, and both survive in the soil for > 30 days (Oregon State University 1996a, 1996b; U.S. EPA. 2005). Pesticide drift can expose rural residents to pesticides without direct occupational contact. For example, measurable concentrations have been detected in the air, in plants, and in animals away from application sites (Chester and Ward 1984; Currier et al. 1982; MacCollom et al. 1986). Outdoor and indoor air concentrations for agriculturally applied pesticides correlate with each other and also correlate with distance to the application sites (Kawahara et al. 2005). Our GIS-derived method of residential exposure estimation has been validated using values determined for organochlorines and measurements of serum biomarkers for dichlorodiphenyldichloroethylene, a metabolite of dichlorodiphenyltrichloroethane (Ritz and Costello 2006).

Direct evidence of any particular pesticide compound contributing to PD in humans is lacking (Brown et al. 2006). This is partially due to a dearth of exposure assessment tools that accurately document past and long-term pesticide exposures in humans. The relatively small effect size any single environmental toxin may exhibit, and the necessity for large sample sizes that allow for an efficient investigation of gene–environment interactions among vulnerable subgroups, may have further hindered progress in this area. The availability of historical pesticide application data for California allowed us to develop a GIS-based method of assessing exposures to pesticides for residents of the highly agricultural California Central Valley. Our study is unique in that a record-based rather than recall-based assessment of historical residential pesticide exposures was possible.

## Conclusion

No previous epidemiologic study has been able to objectively assess general or specific historical residential pesticide exposure in PD; all prior studies relied on subjects’ recall and reporting of pesticide use. We used GIS to combine data from the California PUR and land use maps to identify agricultural pesticide applications in proximity of residences of PD cases and controls. In addition, we assessed occupational pesticide exposures according to job descriptions and self-reported pesticide use. We enrolled more incident, movement disorder specialist–confirmed cases from a high pesticide exposure environment than any study so far. Our genetic findings replicate a prior report and provide additional support for a gene–environment interaction between pesticide exposure and variants of the *DAT* gene.

## Figures and Tables

**Table 1:** Characteristics of the California Central Valley study population.

Characteristic	Cases (*n* = 324) [no. (%)]	Controls (*n* = 334) [no. (%)]	OR (95% CI)
Sex			

Male	179 (55.3)	168 (50.3)	Reference
Female			0.82 (0.60–1.11)

Age (years)^a^			

≤ 60	69 (21.3)	97 (29.0)	Reference
> 60	255 (78.7)	237 (71.0)	1.51 (1.06–2.16)

Race/ethnicity			

White	264 (81.5)	268 (80.2)	Reference
Black	3 (0.93)	12 (3.6)	0.25 (0.07–0.91)
Latino	38 (11.7)	28 (8.4)	1.38 (0.82–2.31)
Asian	3 (0.93)	10 (3.0)	0.31 (0.08–1.12)
Native American	16 (4.9)	16 (4.8)	1.02 (0.50–2.07)

County			

Fresno	162 (50.0)	124 (37.1)	Reference
Kern	99 (30.6)	136 (40.7)	0.56 (0.39–0.79)
Tulare	63 (19.4)	74 (22.2)	0.65 (0.43–0.98)

Education (years)			

0 to < 12	58 (17.9)	34 (10.2)	1.34 (0.79–2.27)
12	89 (27.5)	70 (21.0)	Reference
> 12	177 (54.6)	230 (68.9)	0.61 (0.42–0.88)

First-degree relative with PD			

No	277 (85.5)	299 (89.5)	Reference
Yes	47 (14.5)	35 (10.5)	1.45 (0.91–2.31)

Cigarette smoking			

Never smoker	174 (53.7)	145 (43.4)	Reference
Current smoker	19 (5.9)	33 (9.9)	0.48 (0.26–0.88)
Former smoker	131 (40.4)	156 (46.7)	0.70 (0.51–0.96)

Smoking (pack-years)			

0	174 (53.7)	145 (43.4)	Reference
> 0 to < 10	63 (19.4)	71 (21.3)	0.74 (0.49–1.11)
≥ 10 to < 40	60 (18.5)	81 (24.3)	0.62 (0.41–0.92)
≥ 40	27 (8.3)	37 (11.1)	0.61 (0.35–1.05)

Occupational pesticide exposure^b^			

Not occupationally exposed	206 (63.6)	236 (70.7)	Reference
Possibly exposed	25 (7.7)	24 (7.2)	1.19 (0.66–2.15)
Likely exposed	93 (28.7)	74 (22.2)	1.44 (1.01–2.06)

Residential pesticide exposure to maneb and paraquat			

None/low	286 (88.3)	319 (95.5)	Reference
High	38 (11.7)	15 (4.5)	2.80 (1.52–5.25)

a Age [median (SD)]: Case [70.1(10.4)]; Controls [68.5 (12.5)].

b Job–exposure matrix classification of pesticide exposure was based on occupational codes and self-reported agricultural and ground maintenance pesticide applications.

**Table 2:** Associations between PD and genotypes/diplotypes of the 5′ and 3′ regions of DAT in the present study and Kelada et al. (2006).

	California Central Valley study			Kelada et al. (2006)
Genotype/diplotype	Cases [no. (%)]	Controls [no. (%)]	OR (95% CI)^a^	OR (95% CI)^b^
5′ Clade diplotype				
BB	111 (34.3)	136 (40.7)	Reference	Reference
BA	137 (42.3)	132 (39.5)	1.26 (0.89–1.80)	1.14 (0.81–1.61)
AA	76 (23.5)	66 (19.8)	1.66 (1.08–2.57)	1.40 (0.90–2.20)
3′ VNTR genotype				
10/10	179 (55.3)	200 (59.9)	Reference	Reference
9/10	113 (34.9)	109 (32.6)	1.15 (0.82–1.61)	1.31 (0.95–1.82)
9/9	28 (8.6)	16 (4.8)	1.86 (0.96–3.57)	1.33 (0.74–2.39)
Rare genotypes^c^	4 (1.2)	9 (2.7)	NC	NC

NC, not calculated.

a OR adjusted for sex, smoking (ever/never), age, and race.

b OR adjusted for sex, smoking (ever/never), and age.

c Rare genotypes include 3/3, 3/9, 3/10, 8/10, and 10/11.

**Table 3:** Associations with PD by number of susceptibility alleles^a^ in the 5′ and 3′ regions of DAT the present study and Kelada et al. (2006).

	California Central Valley study			Kelada et al. (2006)
No. of susceptibility alleles	Cases [no. (%)]	Controls [no. (%)]	OR (95% CI)^b^	OR (95% CI)^c^
0	69 (21.3)	85 (25.5)	Reference	Reference
1	109 (33.6)	121 (36.2)	1.03 (0.67–1.56)	1.16 (0.75–1.78)
> 2	146 (45.1)	128 (38.3)	1.50 (1.00–2.25)	1.58 (1.03–2.40)
2	98 (30.3)	102 (30.5)	1.26 (0.82–1.95)	NC
≥ 3	48 (14.8)	26 (7.8)	2.36 (1.31–4.24)	NC
*p*-Value for trend (across all categories)				0.005

NC, not calculated.

a Defined as 5′ A clade and 3′ VNTR 9-repeat alleles [includes genotypes with rare alleles; i.e., a genotype containing a 9 allele (e.g., 3/9) was counted as carrying one susceptibility allele and all others were counted as zero susceptibility alleles].

b OR adjusted for sex, smoking (ever/never), age (continuous), and race.

c OR adjusted for sex and age.

**Table 4:** Associations with PD by number of susceptibility alleles^a^ in the 5′ and 3′ region of DAT and residential paraquat and maneb exposure in the California Central Valley study.

	Residential paraquat and maneb exposure					
	Zero/low			High		
No. of susceptibility alleles	Cases (*n* = 286)	Controls (*n* = 319)	OR^b^ (95% CI)	Cases (*n* = 38)	Controls (*n* = 15)	OR^b^ (95% CI)
0	65	80	Reference	4	5	0.88 (0.22–3.48)
1	99	117	0.98 (0.63–1.52)	10	4	2.99 (0.88–10.21)
≥ 2	122	122	1.30 (0.85–2.00)	24	6	4.53 (1.70–12.09)
*p*-Value for trend (across all categories)						0.0006

a Defined as 5′ A clade and 3′ VNTR 9-repeat alleles [includes genotypes with rare alleles; i.e., a genotype containing a 9 allele (e.g., 3/9) was counted as carrying one risk allele and all others were counted as zero risk alleles].

b OR adjusted for age (continuous), race/ethnicity, education (< 12, 12, > 12 years), smoking (ever/never), and occupational pesticide exposures (job–exposure matrix).

**Table 5:** Associations with PD in males only by number of susceptibility alleles in the 5′ and 3′ region of DAT and occupational pesticide exposure in DAT in the present study and Kelada et al. (2006).

	Unexposed/possibly exposed			Likely exposed		
No. of susceptibility alleles	Cases (*n*)	Controls (*n*)	OR (95% CI)	Cases (*n*)	Controls (*n*)	OR (95% CI)
California Central Valley study: males (*n* = 347)^a^						
0	26	26	Reference	13	18	Reference
1	34	46	0.70 (0.34–1.45)	28	18	2.00 (0.71–5.67)
≥ 2	42	43	0.94 (0.46–1.92)	36	17	2.83 (1.01–7.92)
*p*-Value for trend (across all categories)						0.05
Kelada et al. (2006): males (*n* = 417)^b^						
0	23	37	Reference	7	17	Reference
1	49	60	1.21 (0.62–2.36)	14	23	1.63 (0.52–5.15)
≥ 2	59	87	1.17 (0.62–2.23)	26	15	5.66 (1.73–18.5)

a OR adjusted for age (continuous), race/ethnicity, education (<12, 12, >12 years), smoking (ever/never), and high residential paraquat and maneb pesticide exposure.

b OR adjusted for age (< 60, ≥ 60), education (quintiles), and smoking (ever/never).
